# Antiviral Activity of Total Flavonoid Extracts from *Selaginella moellendorffii* Hieron against Coxsackie Virus B3 *In Vitro* and *In Vivo*

**DOI:** 10.1155/2014/950817

**Published:** 2014-05-19

**Authors:** Dan Yin, Juan Li, Xiang Lei, Yimei Liu, Zhanqiu Yang, Keli Chen

**Affiliations:** ^1^Key Laboratory of TCM Resource and TCM Compound Co-Constructed by Hubei Province and Ministry of Education, Hubei University of Traditional Chinese Medicine, Wuhan 430065, China; ^2^Wuhan Bioengineering Institute, Wuhan 430415, China; ^3^State Key Laboratory of Virology, School of Medicine, Institute of Medical Virology, Wuhan University, Wuhan 430071, China

## Abstract

The antiviral activity of total flavonoid extracts from *Selaginella moellendorffii* Hieron and its main constituents amentoflavone were investigated against coxsackie virus B3 (CVB3). When added during or after viral infection, the extracts and amentoflavone prevented the cytopathic effect (CPE) of CVB3, as demonstrated in a 3-(4,5-dimethylthiazol-2-yl)-2,5-diphenyl tetrazolium bromide (MTT) colorimetric assay, with a 50% inhibitory concentration (IC50) from 19 ± 1.6 to 41 ± 1.2 **μ**g/mL and 25 ± 1.2 to 52 ± 0.8 **μ**g/mL, respectively. KM mice were used as animal models to test the extracts' activity *in vivo*. Oral administration of the total flavonoid extracts at 300 mg/kg/day significantly reduced mean viral titers in the heart and kidneys as well as mortality after infection for 15 days. The experimental results demonstrate that *in vitro* and *in vivo* the model mice infected with CVB3 can be effectively treated by the total flavonoid extracts from *Selaginella moellendorffii* Hieron.

## 1. Introduction

Group B coxsackieviruses have been associated with a wide range of human diseases, such as encephalitis, common cold, cardiomyopathy, diabetes, inflammation, and neurological disorders [1–3]. Among them, CVB3 is the leading cause of viral myocarditis. Because proper infection control practices are still not fully available for CVB3 infection, patients with myocarditis often progress into chronic dilated cardiomyopathy and finally die of heart failure [4]. So far, in China, attempts have been made to incorporate natural herbal medicine into treatment of viral infections [5–7].

*Selaginella moellendorffii* Hieron, a medicinal plant of genus* Selaginella* (Selaginellaceae), has been used in traditional Chinese folk medicine for treatment of jaundice, gonorrhea, bleeding, and acute hepatitis [8]. The primary bioactive constituents of* S. moellendorffii* are reported to be flavones, such as amentoflavone, robustaflavone, biapigenin, hinokiflavone, podocarpusflavone A, and ginkgetin, which have antioxidant, antivirus, and antitumor properties [8–11].

In our previous study, it was found that the total flavonoid extracts of* Selaginella moellendorffii* (TFE) showed strong inhibition effects on CVB3* in vitro* [12]. In this study, the antiviral activity of amentoflavone (the main constituent of* S. moellendorffii*) and TFE against CVB3* in vitro* and* in vivo* is systematically investigated.

## 2. Materials and Methods

### 2.1. Plant Materials and Chemicals

The whole herbs of* Selaginella moellendorffii* Hieron were collected during September 2010 from the Wuduhe town of Yichang (Hubei, China). Identification of specimen was confirmed by Dr. Dingrong Wan, South Central University for Nationalities (Wuhan, China), and a voucher specimen was deposited in the herbarium of Hubei University of Chinese Medicine, China.

Acyclovir (ACV) was produced in Wuhan Changlian Laifu pharmaceutical Limited Liability Company (Wuhan, Hubei, China). RPMI 1640, trypsin, and fetal calf serum (FCS) were purchased from GIBCO (Grand Island, NY, USA). MTT and dimethyl sulfoxide (DMSO) were from Sigma (St. Louis, MO, USA). Deionized water was prepared using a Millipore Milli Q plus system. All other chemicals not mentioned here were of analytical grade from standard sources.

### 2.2. Preparation of Samples

Ethyl acetate extracts of* S. moellendorffii* (EAE): the air-dried and powdered* S. moellendorffii* (50 g) were extracted twice with petroleum ether and then were filtered. The residues were extracted twice with ethyl acetate (500 mL) and then were filtered. Then the solution was dried using a rotary evaporator. The purity of EAE was about 8.9%.

TFE were prepared in our key laboratory according to their preparation technology and quality standard [13, 14]. The content of total flavonoids and the main constituent amentoflavone were not less than 50.0% and 35.0%, respectively. The content of TFE and amentoflavone in EAE was about 17.4% and 5.6%, respectively.

Amentoflavone, as chemical reference substance, was refined from* S. moellendorffii* in the key laboratory with a purity of 99.7% [15, 16].

The above four samples (ACV, EAE, TFE, and amentoflavone) were dissolved in DMSO and stored at −20°C for the assay* in vitro*. Final concentrations for different experiments were prepared by diluting with complete medium. The final maximum DMSO concentration was 0.05%, which showed no effect on cell cultures. The blank control received the carrier solvent. For* in vivo* assay, TFE and ACV were suspended uniformly in distilled water with ultrasonic waves before use.

### 2.3. Cell Cultures and Viruses

HEp-2 (human laryngeal carcinoma) cells and CVB3 were maintained at Institute of Medical Virology, Wuhan University. HEp-2 cells were grown in RPMI 1640 supplemented with 10% fetal calf serum (FCS), 0.1% L-glutamine, 100 U/mL penicillin, and 0.1 mg/mL streptomycin. The test medium used for the cytotoxic assay as well as for antiviral assays contained 2% of FCS in the above medium. The virus was stored in small aliquots at −80°C until use.

### 2.4. Virus Titration

Virus titration was performed by the limited dilution method using a 96-well plate. The virus titer was estimated from cytopathogenicity of cells infected with CVB3 and expressed as 50% tissue culture infectious doses/mL (TCID_50_/mL) [17].

### 2.5. Cytotoxicity and Virus Growth Inhibition Assay

The cytotoxicity and antiviral effect of the samples were determined by quantitative colorimetric MTT method [18, 19]. Briefly, HEp-2 cells (2 × 10^4^/well) were seeded in 96-well plates. After the removal of growth medium, cells were incubated with various concentrations of extract. The plates were incubated at 37°C, and the development of cytopathic effect was monitored daily by light microscopy until the virus-infected, untreated cells showed CPE up to 80%. At this time point, MTT solution (5 mg/mL in phosphate-buffered saline, PBS) was added to each well after the removal of the medium and incubated for an additional 4 h. The formazan crystal formed was dissolved with DMSO. The optical densities (ODs) were then detected at double wavelengths of 540 and 690 nm from the microplate spectrophotometer. The data was expressed as a percentage of OD value of treated cell cultures relative to untreated ones. The 50% cytotoxic concentrations (CC50) and IC50 of the samples were determined. Then, therapeutic index (TI) of the samples by CC50/IC50 was obtained. Each dilution was tested in triplicate.

### 2.6. Treatment of Sample before Virus Infection

The dilutions of the samples were dissolved in RPMI-1640 and incubated with HEp-2 cells in 96-well microtiter plates for 24 h at 37°C in a 5% CO_2_ atmosphere. The cells were washed with PBS and infected with 0.1 mL of CVB3 (100TCID50/mL : 10^−5^) after removal of the samples. The cells were rinsed with PBS after a 1 h incubation and then incubated with test medium until typical CPE was obvious [20]. The inhibition of the virus induced CPE was observed by light microscopy and measured by the MTT assay. Virus controls, normal controls, and ACV controls were included in all assays. All the results of experiments were measured in triplicate.

### 2.7. Virucidal Assay

Viral suspensions were cocultured with the dilutions of the samples at 37°C for 1 h in a 5% CO_2_ atmosphere. Then they were added to the HEp-2 cells in 96-well plates. The solutions containing both samples and viruses were removed after a 1 h incubation and the cells were washed carefully with PBS and then incubated with test medium. The experiment was performed according to the above operation. The antiviral effect was determined using CPE observation and viral titer evaluation following the procedures described above.

### 2.8. Sample Treatment after Virus Infection

The HEp-2 cells were infected with 0.1 mL viruses (100TCID50/mL : 10^−5^) for 1 h at 37°C in a 5% CO_2_ atmosphere. The cells were washed with PBS and added with different doses of the samples. The assay was performed according to the above operation as well.

### 2.9. CVB3 Infections in Mice

Specific-pathogen-free male Kunming mice (10 ± 2 g), obtained from Animal Center of Wuhan University, were used in the study. Sixty mice (10 mice/group, six groups) were used in this experiment. Fifty mice were infected with CVB3 intraperitoneally, and the remaining 10 were used as normal control and injected intraperitoneally with the same volume of PBS. After infection for 24 hours, mice were treated by oral gavage (p.o.) with 0.2 mL of total flavonoids extracts from* S. moellendorffii* at 100, 300, and 900 mg/kg body weight/day, respectively, and treatment was continued daily for 15 days unless otherwise indicated. The mice of ACV control group were administered with ACV orally at 10 mg/kg. The virus and normal control group received water instead of samples.

Three mice from each group were sacrificed by cervical dislocation on day 10 after viral exposure. We recorded the body weights of the mice daily until the animals were killed. The kidneys and hearts were harvested from the mice of each group, which were further homogenized to 10% (weight/volume) suspensions in test medium. The homogenates were frozen and thawed twice to release the virus and then centrifuged at 3,000 rpm for 15 min. Virus titration was determined by the limited dilution method. The remaining animals of each group were observed every day for changes in body weight and for any deaths. We obtained the tissue samples from the kidneys and heart until animals of each group were sacrificed on day 15 after viral exposure for pathological examination (HE staining).

### 2.10. Statistical Analysis

The data were analyzed by SPSS 18.0 software. Results were expressed as mean ± standard deviation for three independent experiments. Survival rates of the mice were compared by Kaplan-Meier Survival analysis (log-rank test). A *P* value of <0.05 was considered statistically significant.

## 3. Results

### 3.1. Cellular Cytotoxicity and Antiviral Potential of Samples from* S. moellendorffii* against CVB3* In Vitro*

ACV, EAE, TFE, and amentoflavone were examined for cytotoxicity and antiviral potential by MTT assay. A cellular toxicity of 85 ± 1.7 *μ*g/mL was recorded for TFE on HEp-2 cells, followed by EAE (60 ± 2.1 *μ*g/mL) and amentoflavone (53 ± 0.9 *μ*g/mL); ACV which was the positive sample had no toxicity in the rage of experimental concentration. The level of cytotoxicity was as follows: ACV < TFE < EAE < amentoflavone (Table 1).

**Figure 1:**
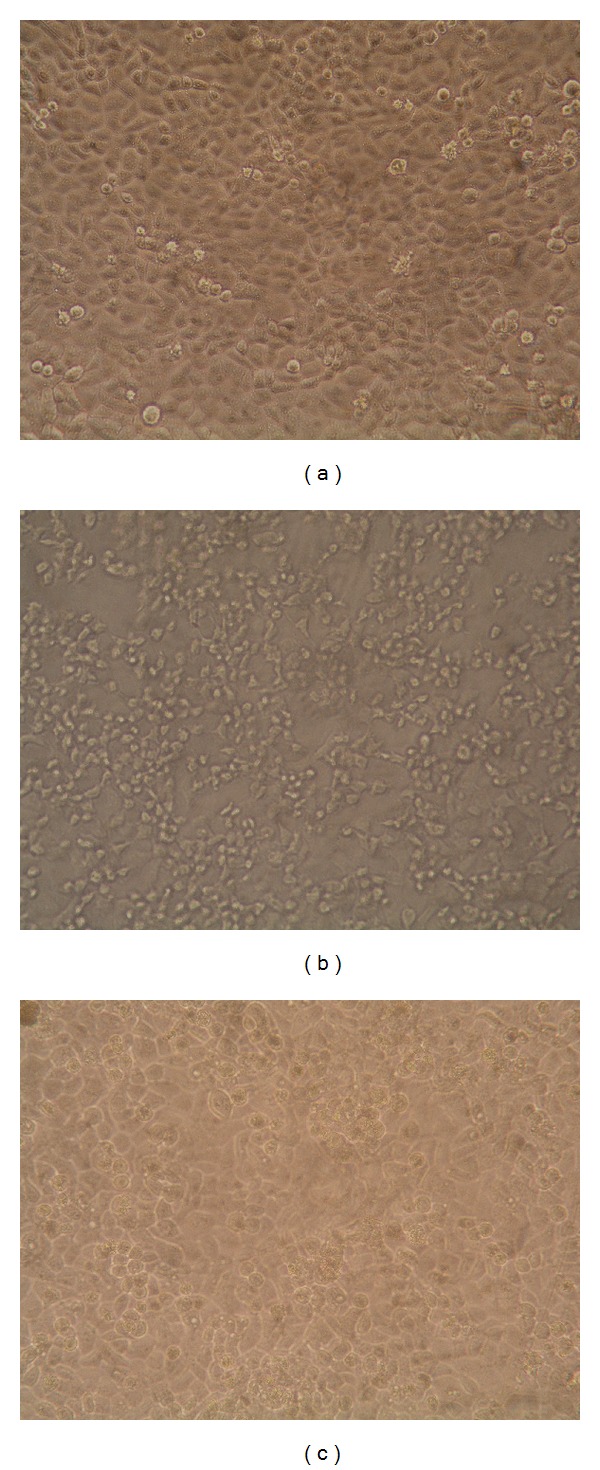
Morphology changes of HEp-2 cells infected with CVB3. (a) Normal control demonstrated that these HEp-2 cells were typical with normal morphology. (b) Viral control demonstrated that most of the HEp-2 cells lost their normal morphology and appeared to be round in shape. Most of them were detached. (c) TFE group demonstrated that some HEp-2 cells had normal morphology and quantity.

Different concentrations of samples from* S. moellendorffii* were subjected to the HEp-2 cells before/after the CVB3 infection or directly mixed with virus as described in Materials and Methods. All samples could not prevent HEp-2 cells from CVB3 infection in the pretreatment assay. There was no significant difference between sample-treated groups and the untreated virus control group, and HEp-2 cells showed typical virus disease which is growth disorders, becoming round or elongated, increasing particles and refraction, and shed or died finally (Figure 1).

**Figure 2:**
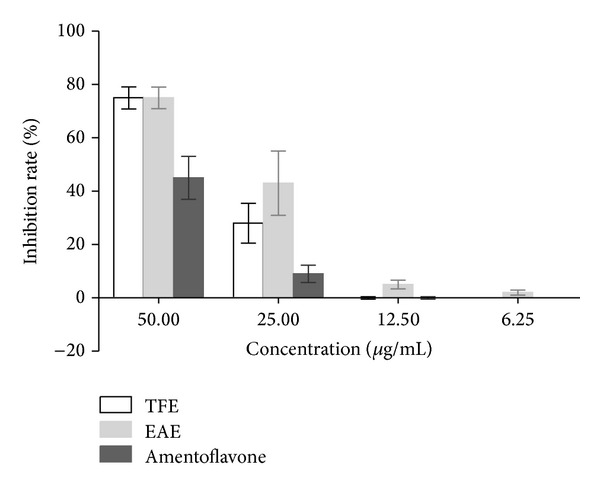
Direct virucidal activity of EAE, TFE, and amentoflavone. The average viral inhibition rate of samples (%) was detected by MTT assay, when they were treated during infection. Viral suspensions were cocultured with the dilutions of the samples (50–6.25 *μ*g/mL) at 37°C for 1 h in a 5% CO_2_ atmosphere.

**Figure 3:**
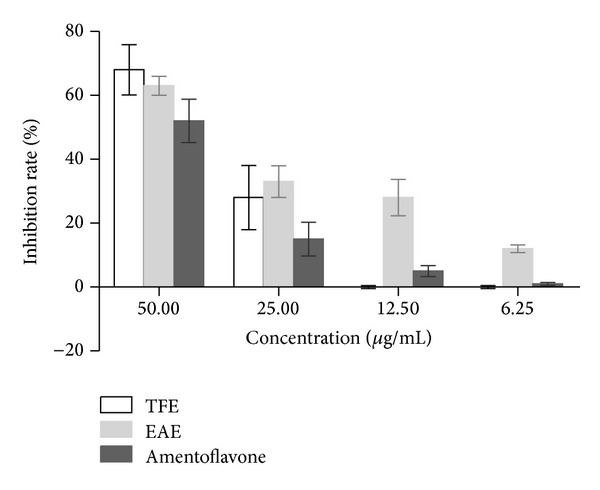
Virucidal activity of EAE, TFE, and amentoflavone after the CVB3 infection. The average viral inhibition rate of samples (%) was detected by MTT assay. The HEp-2 cells were infected with viruses for 1 h at 37°C in a 5% CO_2_ atmosphere. Then cells were washed with PBS and added with different doses of the samples (50–6.25 *μ*g/mL).

However, direct virucidal activity of EAE, TFE, and amentoflavone was observed (Table 1) and the average viral suppression rate of different concentrations of each sample was detected (Figure 2). Also, inhibitory activity of EAE, TFE, and amentoflavone was observed when samples were added after HEp-2 cells had been infected with CVB3 (Table 1) and the average viral suppression rate of various concentrations of each sample was detected (Figure 3). In the assayed concentrations, no virucidal activity of ACV was observed before/after the CVB3 infection or directly mixed with virus.

The level of TI was as follows: TFE > EAE > amentoflavone. There was no significant difference in antiviral activity between EAE and TFE group. However, there was statistically significant difference in amentoflavone group and TFE or EAE group.

### 3.2. Antiviral Effects of TFE against CVB3 in Mice

#### 3.2.1. Clinical Observations

**Figure 4:**
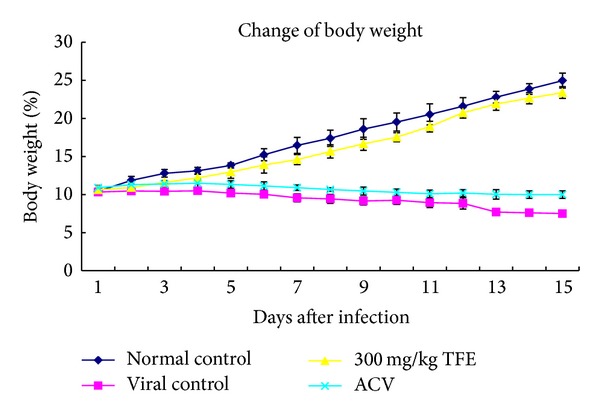
The body weight of CBV3-infected mice during the 15-day period. KM mice were infected intraperitoneally with CVB3 (10^5^TCID_50_/0.2 mL). 24 h after infection, mice were treated by oral gavage with 300 mg/kg/day TFE for 15 days. The virus control group and the normal control group received water instead of the chemicals. Body weight was recorded daily.

In the viral control and ACV group, on day 6 after infection it was observed that animals showed ruffled fur, a tendency to huddle, diminished vitality, and weight loss (Figure 4). On day 7, this group of animals began to die, and, by day 15, all of them had died. In the TFE-treated group, the animals receiving 100 mg/kg/day began to die on day 9 after challenge, and 15-day mortality was 50%. There was no significant difference in body weight between normal control and TFE 300 mg/kg/day group. The animals receiving 300 mg/kg/day and the normal control animals did not show abnormalities during the 15-day period, the body weight of animals was gradually increased in the normal control group (Figure 4), and 15-day mortality reduced to 20%. The 15-day mortality decreased to 10% in mice treated with TFE 900 mg/kg/day (Table 2). The cure rates in mice treated with TFE at the doses of 100, 300, and 900 mg/kg/day were 30%, 50%, and 70%, respectively (Table 2).

#### 3.2.2. Pathological Evaluation

**Figure 5:**
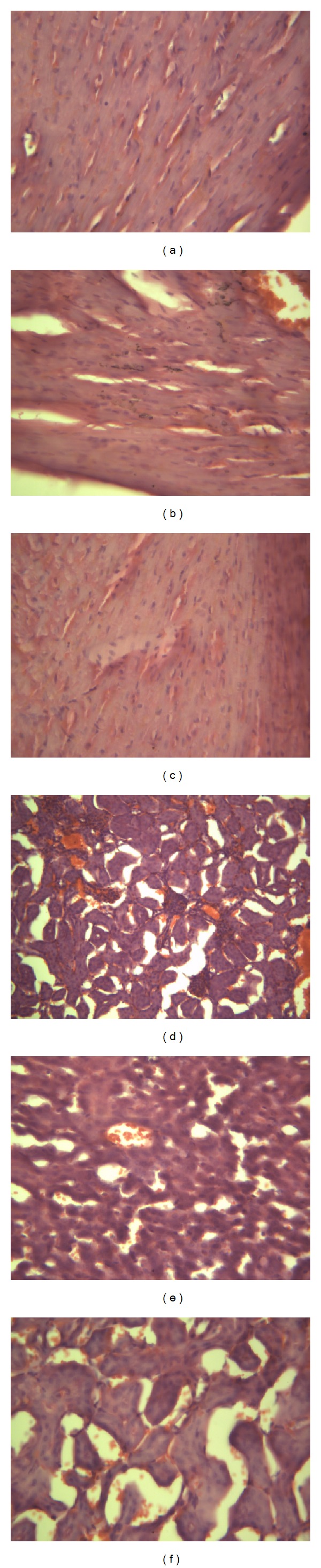
Pathologic features of hearts and kidneys (HE staining, ×200). (a) Normal control of heart tissue; (b) infected control of heart tissue; (c) TFE-treated group of heart tissue; (d) normal control of kidney tissue; (e) infected control of kidney tissue; (f) TFE-treated group of kidney tissue. Animals of each group were sacrificed on day 15 after viral exposure for pathological examination.

We obtained the tissue samples from the kidneys and heart until animals of each group were sacrificed on day 15 after viral exposure for pathological examination. As shown microscopically, inflammatory cell infiltration and glomerular atrophy were in the viral control and ACV group animals, which also showed hyperemia and edema with massive infiltration of the inflammatory cell. Tissue samples from the animals of TFE-treated group did not have obvious changes (Figure 5).

#### 3.2.3. Virological Evaluation

The changes of the virus titers in heart and kidneys of the CVB3-infected mice from different groups at day 10 were shown in Table 3. The virus titers of kidneys and heart were significantly lower in mice receiving oral administration of TFE at three doses for 10 days than in the viral control group and ACV group (*P* < 0.05).

## 4. Discussion

*S. moellendorffii* has been used in traditional Chinese folk medicine for treatment of various inflammatory diseases such as pneumonia, acute tonsillitis, conjunctivitis, mastitis, and icteric hepatitis [8]. In addition,* S. moellendorffii* has been made into medicinal tablets in China, and it has good effect on idiopathic and secondary thrombocytopenic purpura and various bleeding ailments. The chemical constituents of this plant are reported to be biflavonoids, aliphatic acids, sitosterols, coumarins, and lignanosides [21, 22]. EAE contains amentoflavone, hinokiflavone, podocarpusflavone A, robustaflavone, and ginkgetin [8, 16]. Ginkgetin, hinokiflavone, and robustaflavone had antivirus activity, beside amentoflavone [23].

In this study, the antiviral activity of total flavonoids against CVB3* in vitro and in vivo* was investigated. TFE, EAE, and amentoflavone treatments during and after injection exhibit 2~3-fold difference in activities, and the level of TI was as follows: TFE > EAE > amentoflavone; the level of cytotoxicity was as follows: TFE < EAE < amentoflavone. These results showed that single compound with higher cytotoxicity showed relatively weaker antiviral activity than extracts. These indicated that other compounds might have synergistic effect, or the compound showing strong antiviral activity against CVB3 might not be amentoflavone, despite being the main constituent of* S. moellendorffii.* Therefore, further study of amentoflavone* in vivo* was not investigated, and the single compound amentoflavone used in practice might not be a good choice.

When primarily cultured HEp-2 cells infected with CVB3 were treated with TFE by different methods, TFE was found to present potent antiviral activity against CVB3 when added during and after infection. These data demonstrated that TFE could induce durable antiviral activity in host cells, not only inactivating CVB3 but also inhibiting viral replication. Humans medically use biflavonoid especially for antioxidant, anti-inflammatory, and anticarcinogenic. As an antioxidant, amentoflavone inhibits production of NO, which inactivates NF-*κ*B, while, as anti-inflammatory, amentoflavone and ginkgetin inhibit inflammation that induces iNOS and COX-2 at macrophage RAW 264.7. However, the mechanism of antiviral activity needed further study.

The mouse model infected with CVB3 was established to further study whether TFE possessed antiviral action against CVB3* in vivo*. The infected mice display some symptoms of circulatory failure such as cyanosis and lack of blood perfusion in the tails, and there was evidence of virus replication in the heart and kidneys. Pathological findings also confirmed the results, and all viral control mice died on the 15th day after infection. Thus, CVB3 infection in KM mice was a suitable* in vivo* antiviral activity. The mice orally administered TFE 100, 300, or 900 mg/kg/day after infection with CVB3 for 15 days significantly enhanced survival rate compared to the virus group and revealed clear dose-effect relationship. The virus yields were reduced and the pathological abnormalities were released in kidneys and hearts.

ACV, the positive control sample, has been successfully used to treat myocarditis associated with Epstein-Barr and varicella virus, but there was no obvious evidence demonstrating that ACV was effective in CVB3 induced myocarditis.

## 5. Conclusions

In summary, the total flavonoid extracts from* S. moellendorffii* have been found to exhibit an effective antiviral activity against CVB3 infection* in vitro* and* in vivo*. This provides new therapeutic candidates for CVB-induced myocarditis treatment. However, further experiments are required to determine the mechanism and target of action.

## Figures and Tables

**Table 1:** Cytotoxicity (CC50: *μ*g/mL), *in vitro* antiviral activity (IC50: *μ*g/mL), and therapeutic index (TI) for each sample.

Samples	CC50^a^	Treating before infection		Treating during infection		Treating after infection	
		IC50^a^	TI	IC50^a^	TI	IC50^a^	TI
TFE	85 ± 1.7	NA	NA	41 ± 1.2	2.07	19 ± 1.6	4.47
EAE	60 ± 2.1	NA	NA	36 ± 1.9	1.67	16 ± 1.9	3.75
Amentoflavone	53 ± 0.9	NA	NA	52 ± 0.8	1.02	25 ± 1.2	2.12
ACV	>500	NA	NA	NA	NA	NA	NA

CC50: the half of the cytotoxic concentration; IC50: the inhibitory concentration required to reduce viral replication by 50%; TI: the ratio of CC50/IC50; NA: no activity in the assayed concentrations; ACV: acyclovir; EAE: ethyl acetate extracts of *S. moellendorffii*; TFE: total flavonoid extracts of *S. moellendorffii*. ^a^Mean ± SD values are shown from three independent experiments.

**Table 2:** Cure and mortality rate in CBV3-infected mice.

	Cure rate (%)	Mortality rate (%)
Normal control	100	0
Viral control	0	100
TFE-100 mg/kg^b^	30 ± 8**	50 ± 16**
TFE-300 mg/kg^b^	50 ± 12**	20 ± 5**
TFE-900 mg/kg^b^	70 ± 17**	10 ± 12**
ACV-10 mg/kg	0	100

^b^If the body weight of the remaining mice in the TFE-treated group was with no continuous growth, they were considered to be uncured. **P < 0.01, compared with the virus control group.

**Table 3:** Virus titers of heart and kidney in CBV3-infected mice (-lgTCID50).

Groups	Heart	Kidney
Normal control	0	0
Virus control	4.3 ± 0.3	5.3 ± 0.4
TFE-100 mg/kg	3.5 ± 0.3*	3.9 ± 0.3**
TFE-300 mg/kg	1.9 ± 0.2**	2.4 ± 0.1**
TFE-900 mg/kg	1.2 ± 0.1**	1.6 ± 0.2**
ACV-10 mg/kg	4.2 ± 0.2	5.4 ± 0.3

All the viral titers data present in a format of -lgTCID_50_. Values are means ± SD (*n* = 10). *P < 0.05 and **P < 0.01, compared with the virus control group.

## Abbreviations

ACV: Acyclovir
CC50: The half of the cytotoxic concentration
CPE: Cytopathic effect
CVB3: Coxsackievirus B3
DMSO: Dimethyl sulfoxide
EAE: Ethyl acetate extracts of* S. moellendorffii*
FCS: Fetal calf serum
HEp-2: Human laryngeal carcinoma cells
IC50: The inhibitory concentration required to reduce viral replication by 50%
MTT: 3-(4,5-Dimethylthiazol-2-yl)-2,5-diphenyl tetrazolium bromide
ODs: Optical densities
TFE: Total flavonoid extracts of* Selaginella moellendorffii*
TI: Therapeutic index (the ratio of CC50/IC50).
